# ﻿Annotated checklist of Sarcophagidae (Diptera) of Jamaica, with new records

**DOI:** 10.3897/zookeys.1221.135698

**Published:** 2024-12-13

**Authors:** Latoya Foote-Gordon, Eric Garraway, Thomas Pape, Eliana Buenaventura

**Affiliations:** 1 Department of Life Sciences, University of the West Indies, Mona, Kingston, Jamaica University of the West Indies Kingston Jamaica; 2 Natural History Museum of Denmark, Copenhagen, Denmark Natural History Museum of Denmark Copenhagen Denmark; 3 Grupo de Entomología Universidad de Antioquia – GEUA, Universidad de Antioquia, Medellín, Colombia Universidad de Antioquia Medellín Colombia

**Keywords:** Caribbean, checklist, diversity, flesh flies, Jamaica, Miltogramminae, Sarcophaginae, taxonomy

## Abstract

An annotated checklist of the Sarcophagidae of Jamaica is presented based on material collected from 2018 to 2024, supplemented with specimens in museum collections as well as literature records. The checklist comprises 45 species from 21 genera, of which 23 species from 15 genera were collected during the present study and identified based on male terminalia. The following species are recorded from Jamaica for the first time: *Bahamiolaorbitalis* Dodge, Peckia (Sarcodexia) dominicana (Lopes), *Tapacuramariarum* Tibana & Lopes, and Lepidodexia (Harpagopyga) diversipes (Coquillet).

## ﻿Introduction

The family Sarcophagidae or flesh flies is a diverse family of Diptera, currently with 172 genera and 3094 described species (Pape et al. 2011; Buenaventura and Pape 2013), which are classified into three subfamilies: Miltogramminae, Paramacronychiinae, and Sarcophaginae. Members of the family are diverse in their feeding habits, including coprophagy, parasitism, predation and necrophagy (Lopes 1982; Mullen et al. 1984; Ferrar 1987; Bänziger and Pape 2004; Vairo et al. 2015; Buenaventura 2021).

Flesh flies of the large subfamily Sarcophaginae show a variable degree of synanthropy or preference for human-modified environments (Beltran et al. 2012; Yepes-Gaurisas et al. 2013; Valverde-Castro et al. 2017; Buenaventura et al. 2021a), and several species have importance for forensic sciences (Oliveira and Vasconcelos 2010; Segura et al. 2011; Cherix et al. 2012; Szpila et al. 2015; Villet et al. 2017), while others may be mechanical carriers of pathogens (Sukontason et al. 2006) or play a role as general (Howlett et al. 2016) or more specific (Wisniewska et al. 2019) pollinators. Flesh flies are hypothesized to have originated in the Neotropical region (Buenaventura et al. 2021b; Buenaventura 2021; Yan et al. 2021), with species predominantly belonging to the subfamily Sarcophaginae. However, further research is warranted to elucidate their evolutionary history, ecological roles and geographical distribution.

This paper aims to update the list of species of Sarcophagidae from Jamaica based on data obtained from recent collections (2018–2024), specimens in the insect collection in the Department of Life Sciences of the University of the West Indies (**DLSUWI**) and the Natural History Museum of Jamaica (**NHMJ**), and literature records. Research on Sarcophagidae diversity from Caribbean islands has yielded the numbers given in Table 1.

**Table 1.** Sarcophagidae diversity of Caribbean Islands.

Caribbean Island	Number of Species
Antigua (Pape 1996)	1
Barbados (Pape 2024)	1
Cayman Island (Pape 1996)	1
Curaçao (Pape 2024)	1
Guadeloupe (Pape 1996)	2
Grenadines (Pape 1996)	2
St. Lucia (Pape 1996)	3
St. Vincent (Pape 1996)	5
British Virgin Islands (Pape 1996)	4
United States Virgin Islands (Pape 1996)	4
Turks and Caicos Island (Pape 1996)	7
Haiti (Pape 1996)	8
Martinique (Pape 1996)	9
Puerto Rico (Curran 1928)	30
Dominica (Pape 2024)	36
Trinidad & Tobago (Pape 2024)	39
Jamaica (Dodge 1965b; Pape 1989)	39
Bahamas (Dodge 1965a)	43
Cuba (Pape 2024)	55

To date, there are no records of Sarcophagidae species on Caribbean islands such as St. Kitts and Nevis and Grenada. However, the number of flesh fly species documented in the Caribbean archipelago is expected to increase with further field research and more intensive sampling efforts.

Dodge (1965b) provides the most comprehensive documentation of Jamaican Sarcophagidae, recording 39 species, 16 of which were described as new. Few collections or biological observations of Sarcophagidae have been documented from Jamaica since the 1960s (Freeman and Taffe 1974; Freeman and Jayasingh 1975; Pape 1989; Foote 2014; Foote-Gordon and Garraway 2023a, 2023b, 2023c), and the knowledge of Jamaican Sarcophagidae is certainly incomplete.

This research aims to expand the understanding of flesh fly diversity and distribution in Jamaica through comprehensive field collections, a systematic review of historical literature, and the analysis of museum specimens housed at the Natural History Museum of Jamaica and the Department of Life Sciences of the University of the West Indies.

## ﻿Materials and methods

### ﻿Study area

Jamaica is situated in the tropical zone approximately 18 degrees north of the equator and is part of the archipelago of the Caribbean Islands. The island measures 232 km in length, with a width ranging from approximately 48 to 80 km and encompasses an area of 10,992 km^2^ (Wilson 2004).

**Figure 1.**
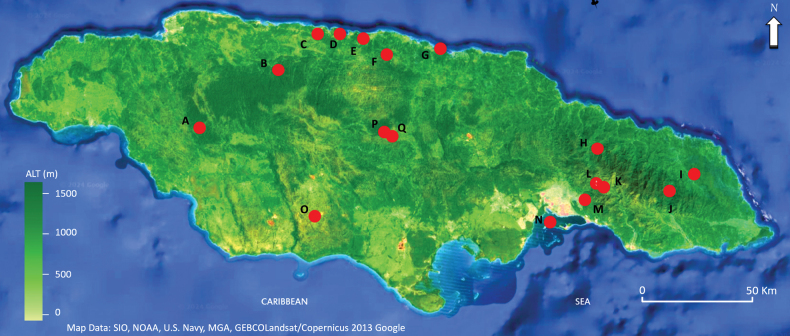
Distribution of sampling sites in Jamaica between 2018 and 2024. **A** Merrywood, St. Elizabeth; **B** Windsor, Trelawny; **C, D** Rio Bueno, St. Ann; **E** Belair, St. Ann; **F** Green Grotto, St. Ann; **G** Roaring River, St. Ann; **H** Hardware Gap, Portland; **I** Comfort Castle, Portland; **J** Bowden Pen, St. Thomas; **K** Salt Hill, St. Andrew; **L** Red Light, St. Andrew; **M** Mona, St. Andrew; **N** Port Royal, St. Andrew; **O** Newport, Manchester; **P, Q** Mason River, Clarendon.

The study area encompasses twelve habitat types (Table 2), such as coastal and freshwater mangrove forests, dry and wet limestone forests, wet and dry forests, wet and dry montane forests, inland wetlands, urban and suburban communities, and rural farms. A total of 17 sampling localities were selected across these habitat types (Fig. 1, Table 2).

**Table 2.** List of sampling localities of Sarcophagidae in Jamaica between 2018 and 2024.

	Locality	Geographic coordinates	Altitude (m)	Habitat description
**A**	Merrywood, St. Elizabeth	18°13'04"N, 77°51'02"W	220	Rural farm
**B**	Windsor, Trelawny	18°21'09"N, 77°38'47"W	98	Wet limestone forest
**CD**	Rio Bueno property, St. Ann	18°28'30"N, 77°26'41"W	25	Dry limestone forest
		18°28'01"N, 77° 27'51"W	10	Solitary wasp nest
**E**	Belair, St. Ann	18°27'23"N, 77°21'08"W	15	Dry limestone forest
**F**	Green Grotto, St. Ann	18°05'15"N, 77°24'57"W	15	Freshwater mangrove forest
**G**	Roaring River, St. Ann	18°24'52"N, 77°09'32"W	94	Dry limestone forest
**H**	Hardware Gap, Portland	18°05'15"N, 76°42'13"W	1050	Wet montane forest
**I**	Comfort Castle, Portland	18°03'14"N, 76°24'46"W	147	Wet montane forest, rural/farm community
**J**	Bowden Pen, St. Thomas	18°02'27"N, 76°23'55"W	290	Wet limestone forest
**K**	Salt Hill, St. Andrew	18°02'00"N, 76°40'29"W	1210	Dry montane forest and farmlands
**L**	Red Light, St. Andrew	18°03'36"N, 76°43'23"W	988	Dry forest and suburban community
**M**	Mona, St. Andrew	18°00'22"N, 76°45'00"W	180	Urban community
**N**	Port Royal, St. Andrew	17°56'29"N, 76°50'02"W	4	Coastal mangrove forest
**O**	Newport, Manchester	17°57'17"N, 77°29'41"W	715	Suburban community
**PQ**	Mason River, Clarendon	18°11'47"N, 77°15'35"W	700	Inland wetland

#### ﻿Specimen sampling, identification, and documentation

Field expeditions were carried out between 2018 and 2024. Sample collection was conducted throughout the year, regardless of rainy and dry seasons, depending on the availability of resources. Many of the flies were collected with Van Someren-Rydon (VSR) traps and plastic bottle traps (Hwang and Turner 2005), and a few were collected with hand nets. Traps were baited separately with various decomposing meats, such as chicken and pork, and fermented fruits. At each site, two VSR traps were placed at a minimum height of 1.5 m above ground and spaced at least 50 m apart. The traps were left in place for a minimum of 4 h and a maximum of 12 h.

Specimens were collected and preserved in 95% ethanol. Flesh flies were carefully pinned, and their terminalia extended for detailed examination and taxonomic identification. Taxonomic identifications were made of males only, as females are difficult to identify. Taxonomic keys, descriptions, and illustrations by Dodge (1965a, b), Giroux and Wheeler (2009), and Buenaventura and Pape (2013) were used to identify species.

Neotropical distribution data were taken from ‘A taxonomic database to all flesh flies’ (Pape 2024), and distribution in Jamaica is based on the specimens collected during the present study and specimens from the insect collections of the Department of Life Sciences, University of the West Indies (**DLSUWI**) and the Natural History Museum of Jamaica (**NHMJ**).

Photographs of male terminalia were produced with a Leica M205 C stereo microscope system camera.

### ﻿Format of checklist

The checklist is arranged in alphabetical order, first by subfamily, then by genus and species. Each species entry starts with a valid species name, the authority, and the year of publication. For all collected specimens and museum material, the following information is recorded: locality and date of collection, number and sex of specimens, collector(s), and depository. Entries are separated by semicolons. For localities with multiple hierarchical levels, a comma separates the exact sampling site from the main locality or parish. Species previously recorded from Jamaica have their published records listed in a section titled “Literature records”, while species recorded from Jamaica for the first time are indicated as “New records.” For each species, the general distribution within the Neotropical region is also provided. Remarks are included when applicable.

## ﻿Results

A total of 731 specimens of flesh flies from Jamaica were examined from field expeditions, which included 325 females and 406 males, with 45% of the males belonging to only four species (Table 3). The survey revealed new records of flesh flies for the island, namely *Bahamiolaorbitalis* Dodge, 1965, Peckia (Sarcodexia) dominicana Lopes, 1982, *Tapacuramariarum* Tibana & Lopes, 1985, and Lepidodexia (Harpagopyga) diversipes (Coquillet, 1900). These new records increased the total number of flesh fly species known from the country to 45 (Table 4). Most of the species belong to the genus *Peckia* Robineau-Desvoidy, 1830 with six species, followed by *Oxysarcodexia* Townsend, 1917 with three species. The remaining 13 genera are represented by one or two species each. The rarest species found within the genus *Peckia* are Peckia (Euboettcheria) buethni (Dodge, 1965) and Peckia (Peckia) hillifera (Aldrich, 1916), each with only one individual, found in Rio Bueno, St. Ann and in Belair, St. Ann, respectively.

**Table 3.** Abundance and distribution of the most common and widespread species during the study. Only males are included.

Species	Number of individuals	Localities
*Bahamiolaorbitalis*	94	5
*Oxysarcodexiapeltata*	46	10
*Peckiachrysostoma*	29	8
*Peckianicasia*	14	6

### ﻿Checklist

#### ﻿Subfamily Miltogramminae Lioy, 1864

**Genus *Amobia* Robineau-Desvoidy**

**1. *Amobiafloridensis* (Townsend, 1892)**

**Literature records.**Dodge (1965b); Lopes (1969); Freeman and Taffe (1974); Freeman and Jayasingh (1975); Pape (1996).

**Neotropical distribution.** Belize, Brazil, Costa Rica, Cuba, Ecuador, Galápagos Is, Guyana, Jamaica, Panama, Peru, Puerto Rico, Trinidad & Tobago, Venezuela.

##### Genus *Metopia* Meigen

**2. *Metopiaargyrocephala* (Meigen, 1824)**

**Literature records.**Johnson (1919, as *Metopialeucocephala*); Gowdеy (1926); Dodge (1965b); Lopes (1969); Pape (1996).

**Neotropical distribution.** Belize, Cuba, Dominican Republic, Ecuador, El Salvador, Guatemala, Jamaica, Mexico, Peru, Puerto Rico.

**Newly collected** material. • Rio Bueno Property, St. Ann; 31 May 2018; 1 ♂; E. Buenaventura leg. (DLSUWI).

**Remarks.** Collected during the present study with a sweep net near nests of solitary wasps.

##### Genus *Opsidia* Coquillett

**3. *Opsidiajamaica* Pape, 1989**

**Literature records.**Pape (1989); Pape (1996).

**Neotropical distribution.** Jamaica.

##### Genus *Senotainia* Macquart

**4. *Senotainiarubriventris* Macquart, 1846**

**Literature records.**Johnson (1919); Dodge (1965b); Lopes (1969); Pape (1996).

**Neotropical distribution.** Bahamas, Jamaica, Puerto Rico.

**5. *Senotainiatrilineata* (Wulp, 1890)**

**Literature records.**Johnson (1919); Dodge (1965b); Lopes (1969); Pape (1996).

**Neotropical distribution.** Bahamas, Costa Rica, El Salvador, Jamaica, Mexico, Nicaragua, Peru.

#### ﻿Subfamily Sarcophaginae Macquart, 1834

##### Genus *Argoravinia* Townsend, 1917

**6. *Argoraviniacandida* (Curran, 1928)**

**Literature records.**Dodge (1965b); Lopes (1969); Pape (1996); Carvalho-Filho and Esposito (2012).

**Neotropical distribution.** Cuba, Jamaica, Puerto Rico.

**7. *Argoraviniarufiventris* (Wiedemann, 1830)**

**Literature records.**Dodge (1965b, as *Argoraviniamodesta*); Lopes (1969); Pape (1996); Livingstone (2006); Dufek et al. (2015); Sousa et al. (2015).

**Neotropical distribution.** Argentina, Brazil, Colombia, Jamaica, Trinidad & Tobago.

**Newly collected** material. • Mona, St. Andrew; 06 Sep. 2018; 46 ♂; L. Foote leg. (DLSUWI).

**Museum material.** • Rio Cobre, St. Catherine; 23 Sept. 1954; 1 ♂; T. H. Farr leg. (NHMJ).

**Remarks.** Found associated with corpses, hence of potential forensic importance (Dufek et al. 2015). It is known to infest turtle eggs (Smith 2001; Livingstone 2006). In the current study, it was collected in VSR traps containing decomposing chicken. It has been collected from the carcasses of bears, deer and swine in Louisiana using pitfall traps and manual sampling (Grindley-Watson 2004). *Argoraviniarufiventris* is associated with human faeces, fish and bovine spleen (Barbosa 2019). It is also collected from pig carcasses (Barros et al. 2008).

##### Genus *Bahamiola* Dodge, 1965

**8. *Bahamiolaorbitalis* Dodge, 1965**

**Neotropical distribution.** Bahamas, Jamaica (New record).

**Newly collected material.** • Windsor, Trelawny; 01 Jun. 2018; 38 ♂; L. Foote and E. Buenaventura leg. (DLSUWI) • Green Grotto, St. Ann, 31 May 2018; 19 ♂; L. Foote and E. Buenaventura leg. (DLSUWI) • Belair, St. Ann; 31 May 2018; 16 ♂; L. Foote and E. Buenaventura leg. (DLSUWI) • Rio Bueno Property, St. Ann; 31 May 2018; 2 ♂; L. Foote and E. Buenaventura leg. (DLSUWI) • Red Light, St. Andrew; 19 Mar. 2024; 19 ♂; L. Foote leg. (DLSUWI).

**Remarks.** The genus contains two species, *Bahamiolaorbitalis* and *Bahamiolagregori* Rohdendorf, 1971. This study presents the first record of the genus and species in Jamaica. It was collected in VSR traps with decomposing chicken and was the most frequently collected species during the study.

##### Genus *Blaesoxipha* Loew, 1861

**9. Blaesoxipha (Kellymyia) jamacoorum (Dodge, 1965)**

Fig. 2

**Figure 2.**
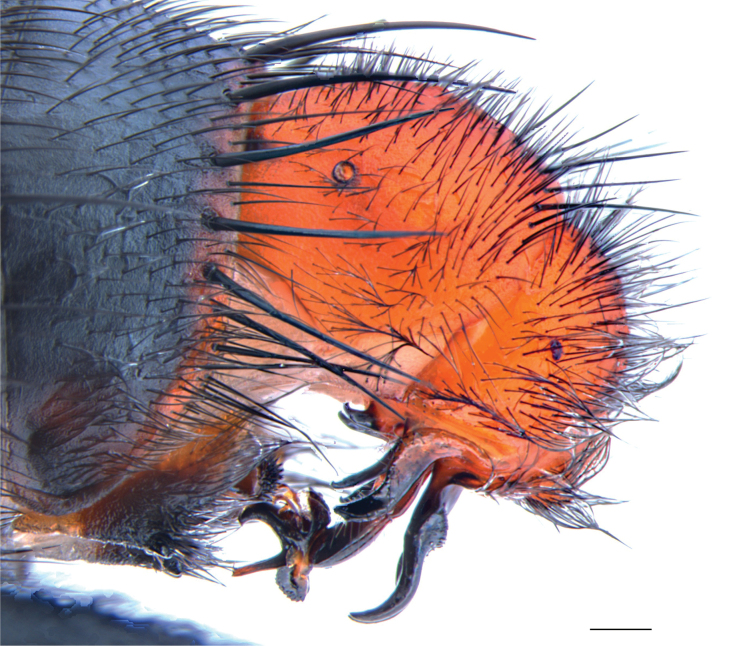
Blaesoxipha (Kellymyia) jamacoorum. Male terminalia, lateral view; endemic to Jamaica. Scale bar: 1 mm.

**Literature records.**Dodge (1965b); Lopes (1969); Pape (1996).

**Neotropical distribution.** Jamaica.

**Newly collected material.** • Roaring River, St. Ann; 19 Oct. 2018; 2 ♂; L. Foote leg. (DLSUWI) • Mason River, Clarendon; 26 Nov. 2019; 1 ♂; L. Foote leg. (DLSUWI) • Red Light, St. Andrew; 19 Mar. 2024; 3 ♂; L. Foote leg. (DLSUWI).

**Museum material.** • Mocho, Clarendon; 16 Nov. 1978; 1 ♂; J. Simpson leg. (DLSUWI) • Jacksonville; 05 Oct 1997; 1 ♂; M. Peddie leg. (DLSUWI) • Highgate, St. Mary; 05 Oct. 2008; 1 ♂; M. Grant leg. (DLSUWI) • Windsor, Trelawny; 28 Sep. 2014; 1 ♂; D. Wilkins leg. (DLSUWI) • Lewisburg, St. Mary; 18 Oct. 2015; 1 ♂; Heslop leg. (DLSUWI) • Halse Hall, Clarendon; Mona, St. Andrew; 27 Oct. 2016; 2 ♂; K. Minott leg. (DLSUWI).

**Remarks.** Collected on overripe mango fruit by Dodge (1965b). In this study, it was collected from decomposing chicken and pork.

**10. Blaesoxipha (Gigantotheca) plinthopyga (Wiedemann, 1830)**

**Literature records.**Johnson (1919); Lopes (1941); Dodge (1965b); Pape (1996); Mello-Patiu (2016).

**Neotropical distribution.** American Virgin Is, Bahamas, Brazil, Costa Rica, Cuba, Dominica, Dominican Republic, El Salvador, Galápagos Is, Guatemala, Guyana, Jamaica, México, Nicaragua, Panamá, Puerto Rico, Venezuela.

**Newly collected material.** • Mona, St. Andrew; 26 Jun. 2018; 10 ♂; L. Foote leg. (DLSUWI).

**Museum material.** • Morant Bay, St. Thomas; 28 Jan. 1989; 1 ♂; (DLSUWI) • Stony Hill, St. Andrew; 17 May 1992; 1 ♂; J. Rodent leg. (DLSUWI) • Meadowbrook Estate, Kingston; 21 Oct 2003; 1 ♂; C. McIntosh leg. (DLSUWI) • Spanish Town, St. Catherine; 18 Nov. 2006; 1 ♂; T. McIntyre leg. (DLSUWI) • Havendale, Kingston; 09 Nov. 2011; 1 ♂; P. Sutherland leg. (DLSUWI) • Mona, St. Andrew; 17 Mar. 2015; 2 ♂; Gilles-Lee leg. (DLSUWI) • Downtown, Kingston; 07 Nov. 1946; 2 ♂, 5 ♀; G. B. Thomspon leg. (NHMJ) • Downtown, Kingston; 18 Dec. 2013; 6 ♂, 4 ♀; L. Wright leg. (NHMJ).

**Remarks.** This widely distributed species was reported on a human corpse in the USA (Wells and Smith 2013), and it is considered medically and forensically important (Barbosa 2019). Dodge (1965b) mentions specimens that were “bred from dead crocodile.” During the study period, it was reared from buried pork bait in Jamaica.

##### Genus *Boettcheria* Parker, 1914

**11. *Boettcheriaparkeri* (Aldrich, 1916)**

Fig. 3

**Figure 3.**
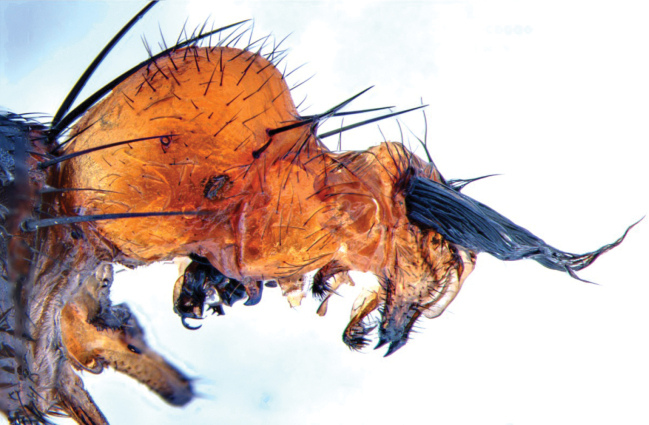
*Boettcheriaparkeri*. Male terminalia, lateral view; endemic to Jamaica. Scale bar: 1 mm.

**Literature records.**Johnson (1919); Dodge (1965b); Lopes (1969); Pape (1996).

**Neotropical distribution.** Jamaica.

**Newly collected material.** • Bowden Pen, St. Thomas; 05 Jun. 2018; 7 ♂; E. Buenaventura leg. (DLSUWI) • Salt Hill, St. Andrew; 26 Feb. 2024; 1 ♂; L. Foote leg. (DLSUWI).

**Museum material.** • Hermitage Reservoir, St. Andrew; 30 May 1954; 2 ♂; T. H. Farr leg. (NHMJ) • Corn Puss Gap, St. Thomas; 04 Aug. 1948; 1 ♂; R. P. Bengry leg. (NHMJ) • Unity Valley, St. Ann; 14 Nov. 1954; 1 ♂; T. H. Farr leg. (NHMJ).

**Remarks.***Boettcheriaparkeri* is still the only species of *Boettcheria* known from Jamaica (Pape 1996), and as for most other species in this genus, the biology is unknown. It was collected in a VSR trap baited with decomposing chicken and pork. Members of the genus are often listed as carrion flies (Ramírez-Mora et al. 2012) and are found in a variety of habitats, from old-growth forests to urban areas (Dahlem and Downes 1996).

##### Genus *Chrysagria* Townsend

**12. *Chrysagriaduodecimpunctata* Townsend, 1935**

**Literature records.**Dodge (1965b, as *Sarcofahrtiamyiatenta*); Lopes (1969, as *Sarcofahrtiamyiatenta*); Pape (1996); Mello-Patiu (2016).

**Neotropical distribution.** Argentina, Brazil, Colombia, Dominica, Ecuador, Guatemala, Jamaica, Mexico, Peru.

##### Genus *Dexosarcophaga* Townsend, 1917

**13. *Dexosarcophagaruthae* (Dodge, 1965)**

**Literature records.**Dodge (1965b); Lopes (1969); Mello (1996); Pape (1996).

**Neotropical distribution.** Jamaica.

**Remarks.** The type series was collected “over broken nest of *Nasutitermes*” (Dodge 1965b).

##### Genus *Helicobia* Coquillett, 1895

**14. *Helicobiamorionella* (Aldrich, 1930)**

**Literature records.**Dodge (1965b); Lopes (1969); Pape (1996); Mello-Patiu (2016); Dufek (2019); Dufek et al. (2020).

**Neotropical distribution.** American Virgin Is, Argentina, Bahamas, Brazil, Colombia, Costa Rica, Cuba, Dominica, Ecuador, El Salvador, Guatemala, Haití, Jamaica, México, Puerto Rico, Venezuela.

**Newly collected material.** • Rio Bueno Property, St. Ann; 31 May 2018; 1 ♂; L. Foote and E. Buenaventura leg. (DLSUWI) • Belair, St. Ann; 31 May 2018; 1 ♂; L. Foote and E. Buenaventura leg. (DLSUWI).

**Museum material.** • Windsor Hotel, St. Ann; 19 Mar. 1955; 1 ♂; T. H. Farr leg. (NHMJ) • Ferry, St. Andrew, 30 Oct. 1946; 1 ♂; G. B. Thompson leg. (NHMJ) • Ferry, St. Andrew; 03 Oct. 1954; 2 ♂; T. H. Farr leg. (NHMJ) • Mona, St. Andrew; 20 Jan. 1947; 2 ♂; G. B. Thompson leg. (NHMJ).

**Remarks.** This necrophagous species is considered to be of forensic importance (Early and Goff 1986). It was collected from a decomposing crab and decomposing chicken in the present study.

**15. *Helicobiarapax* (Walker, 1849)**

**Literature records.**Johnson (1919, as *Sarcophagahelicis*); Dodge (1965b); Lopes (1969); Pape (1996); Mello-Patiu (2016).

**Neotropical distribution.** Argentina, Belize, Brazil, Cuba, Dominica, Ecuador, El Salvador, Jamaica, Martinique, Mexico, Panama, Peru, Puerto Rico.

**Museum material.** • Road to Holly Mount, St. Andrew; 24 Sept. 1954; 1 ♂; R. P. Bengry leg. (NHMJ) • Mona, St. Andrew; 30 Jan. 1947; 1 ♂; G. B. Thompson leg. (NHMJ) • Half Way Tree, St. Andrew; 06 Aug. 1950; 1 ♂; R. B. Bengry leg. (NHMJ) • Troy, Trelawny; 25 Sept. 1954; 1 ♂; T. H. Farr leg. (NHMJ).

##### Genus *Lepidodexia* Brauer & Bergenstamm, 1891

**16. Lepidodexia (Harpagopyga) albihirta (Dodge, 1965)**

**Literature records.**Dodge (1965b); Lopes (1969); Pape (1996).

**Neotropical distribution.** Jamaica.

**17. Lepidodexia (Harpagopyga) atrata (Dodge, 1965)**

**Literature records.**Dodge (1965b); Lopes (1969); Pape (1996).

**Neotropical distribution.** Jamaica.

**18. Lepidodexia (Harpagopyga) dissimilis (Dodge, 1965)**

**Literature records.**Dodge (1965b); Lopes (1969); Pape (1996)

**Neotropical distribution.** Jamaica.

**19. Lepidodexia (Harpagopyga) diversipes (Coquillet, 1900)**

**Neotropical distribution.** Cuba, Puerto Rico, Jamaica (New record).

**Museum material.** Hardware Gap, Portland; 27 Jul. 1949; 1 ♂; C. B. Lewis leg. (NHMJ).

**20. Lepidodexia (Harpagopyga) nigribimbo (Dodge, 1965)**

**Literature records.**Dodge (1965b); Lopes (1969); Pape (1996).

**Neotropical distribution.** Jamaica.

**21. Lepidodexia (Harpagopyga) villipes (Dodge, 1965)**

**Literature records.**Dodge (1965b); Lopes (1969); Pape (1996).

**Neotropical distribution.** Jamaica.

##### Genus *Oxysarcodexia* Townsend, 1917

**22. *Oxysarcodexiabakeri* (Lopes, 1945)**

**Literature records.**Dodge (1965b); Lopes (1969); Pape (1996); Mello-Patiu (2016); Souza et al. (2020).

**Neotropical distribution.** Bahamas, Brazil, Chile, Colombia, Cuba, Dominica, Ecuador, El Salvador, Galápagos Is, Guadeloupe, Haití, Honduras, Jamaica, México, Panamá, Puerto Rico, Turks & Caicos Is, Venezuela.

**Newly collected material.** • Belair, St. Ann; 31 May 2018; 1 ♂; L. Foote and E. Buenaventura leg. (DLSUWI) • Green Grotto, St. Ann; 31 May 2018; 1 ♂; L. Foote and E. Buenaventura leg. (DLSUWI) • Merrywood, St. Elizabeth; 24 May 2021; 1 ♂; R. Daley leg. (DLSUWI) • Newport, Manchester; 18 Aug. 2023; 1 ♂; R. Daley leg. (DLSUWI) • Red Light, St. Andrew; 20 Mar. 2024; 1 ♂; L. Foote leg. (DLSUWI) • Comfort Castle, Portland; 27 Mar. 2024; 1 ♂; L. Foote leg. (DLSUWI).

**Museum material.** • Cross Roads, St. Andrew; 05 Sep. 1954; 2 ♀, 1 ♂; T. H. Farr leg. (NHMJ) • Rio Cobre, St. Catherine; 28 Feb. 1954; 1 ♀; T. H. Farr leg. (NHMJ) • Negril, Westmoreland; 19 Jul. 1954; 1 ♀; T. H. Farr leg. (NHMJ) • Molland Bay, St. Thomas; 28 Nov. 1954; 1 ♂; T. H. Farr leg. (NHMJ) • Swamp, St. Thomas; 04 Feb. 1955; 1 ♂; T. H. Farr leg. (NHMJ) • Chovey House, St. Mary; 12 Sept. 1954; 1 ♂; T. H. Farr leg. (NHMJ) • Discovery Bay, St. Ann; 11 Nov. 2012; 1 ♂; Wisdom leg. (DLSUWI) • Woodford, St. Andrew; 08 Nov. 2013; 1 ♂; T. Barrett leg. (DLSUWI) • Windsor, Trelawny; 31 Oct. 2015; 2 ♂; E. Reid leg. (DLSUWI).

**Remarks.** Ubiquitous species with a preference for human settlements (Yepes-Gaurisas et al. 2013). Reports of coprophagous (Flores and Dale 1995) and necrophagous (Yepes-Gaurisas et al. 2013) habits.

**23. *Oxysarcodexiachaetopygialis* (Williston, 1896)**

**Literature records.**Dodge (1965b); Lopes (1969); Pape (1996); Souza et al. (2020).

**Neotropical distribution.** Jamaica, St. Vincent.

**24. *Oxysarcodexiacorolla* Dodge, 1965**

**Literature records.**Dodge (1965b); Lopes (1969); Pape (1996); Souza et al. (2020).

**Neotropical distribution.** Jamaica.

**Newly collected material.** • Hardware Gap, Portland; 29 May 2018; 5 ♂; L. Foote and E. Buenaventura leg. (DLSUWI) • Bowden Pen, St Thomas; 05 Jun. 2018; 2 ♂; L. Foote and E. Buenaventura leg. (DLSUWI) • Red Light, St. Andrew; 26 Feb. 2024; 1 ♂; L. Foote leg. (DLSUWI).

**Remarks.** Little is known about the species except its morphology described by Dodge (1965b). Specimens were collected in a VSR trap baited with decomposing chicken in this study.

**25. *Oxysarcodexiadorisae* Dodge, 1965**

**Literature records.**Dodge (1965b); Lopes (1969); Pape (1996); Souza et al. (2020).

**Neotropical distribution.** Jamaica.

**26. *Oxysarcodexiapeltata* (Aldrich, 1916)**

**Literature records.**Johnson (1919); Dodge (1965b); Lopes (1946, 1969); Pape (1996); Souza et al. (2020).

**Neotropical distribution.** Bahamas, Cuba, Dominica, Guadeloupe, Jamaica, Mexico, Panama, Puerto Rico, San Andres Islands, St. Lucia, St. Vincent.

**Newly collected material.** • Green Grotto, St. Ann; 31 May 2018; 11 ♂; L. Foote and E. Buenaventura leg. (DLSUWI) • Belair, St. Ann; 31 May 2018; 7 ♂; L. Foote and E. Buenaventura leg. (DLSUWI) • Windsor, Trelawny; 01 Jun. 2018; 5 ♂; L. Foote and E. Buenaventura leg. (DLSUWI) • Bowden Pen, St. Thomas; 05 Jun. 2018; 2 ♂; L. Foote and E. Buenaventura leg. (DLSUWI) • Hardware Gap, Portland; 29 May 2018; 3 ♂; L. Foote and E. Buenaventura leg. (DLSUWI) • Merrywood, St. Elizabeth; 24 May 2021; 2 ♂; R. Daley leg. (DLSUWI) • Newport, Manchester; 18 Aug. 2023; 2 ♂; R. Daley leg. (DLSUWI) • Red Light, St. Andrew; 20 Feb. 2024; 1 ♂; L. Foote leg. (DLSUWI) • Comfort Castle, Portland; 27 Mar. 2024; 10 ♂; L. Foote leg. (DLSUWI).

**Museum material.** • 4 miles South of Buff Bay, Portland; 14 Mar. 1947; 1 ♂; G. B. Thompson leg. (NHMJ) • Quickstep, Trelawny; 10 Mar. 1949; 1 ♀; C. B. Lewis leg. (NHMJ) • Hermitage Dam, St. Andrew; 21 Jan. 1947; 1 ♂; C. B. Lewis leg. (NHMJ) • Negril, Westmoreland; 19 Jul. 1954; 1 ♀; T. H. Farr leg; (NHMJ) • Whitfield Hall, St. Thomas; Dec. 1954; 1 ♀; G.R. Proctor leg. (NHMJ) • Ferry, St. Andrew; 03 Oct. 1954; 2 ♂; T. H. Farr leg. (NHMJ) • Beverly Hills, St. Andrew; 26 Dec. 1954; 1 ♀; (NHMJ) • Long Mountain, St. Andrew; 19 Sep. 1954; 2 ♀, 1 ♂; T. H. Farr leg. (NHMJ) • Rock Hall, St. Andrew; 17 Oct. 1984; 1 ♂; P. Coward leg. (DLSUWI) • Hope Gardens, St. Andrew; 09 Nov. 2003; 2 ♂; V. Thompson leg. (DLSUWI) • Spanish Town, St. Catherine; 02 Nov. 2011; 1 ♂; K. Reid leg. (DLSUWI) • Mona, St. Andrew; 10 Apr. 2014; 2 ♂; S. Matthew leg. (DLSUWI) • Discovery Bay, St. Ann; 14 Sep. 2014; 2 ♂; J. Dixon leg. (DLSUWI) • Roaring River, St. Ann; 03 Oct. 2014; 1 ♂; S. McKenzie leg. (DLSUWI).

**Remarks.** Known for its role as a pollinator of the White Mangrove, *Lagunculariaracemosa* (Sánchez-Núñez and Mancera-Pineda 2012). It was collected from decomposing chicken during the present study. *Oxysarcodexiapeltata* was the second most frequently collected species during the sampling period, with a presence confirmed across ten localities.

##### Genus *Peckia* Robineau-Desvoidy, 1830

**27. Peckia (Euboettcheria) buethni Dodge, 1965**

Fig. 4

**Figure 4.**
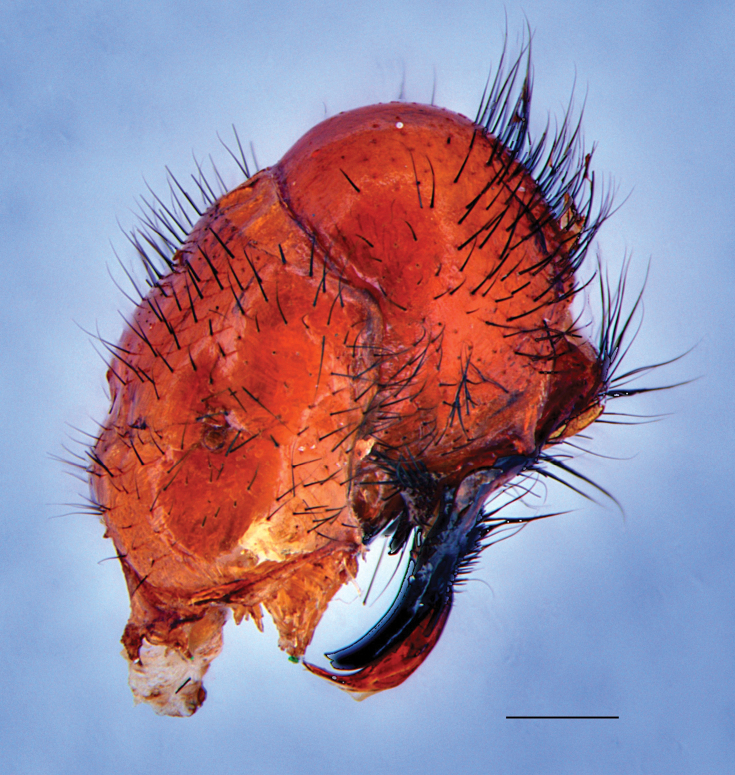
Peckia (Euboettcheria) buethni. Male terminalia, lateral view; endemic to Jamaica. Scale bar: 1 mm.

**Literature records.**Dodge (1965b); Lopes (1969); Pape (1996); Buenaventura and Pape (2013).

**Neotropical distribution.** Jamaica.

**Newly collected material.** Rio Bueno Property, St. Ann; 31 May 2018; 1 ♂; L. Foote and E. Buenaventura leg. (DLSUWI).

**Remarks.** This species is only known from Jamaica. Dodge (1965b) first described it from Papine, Kingston, approximately 114 km from its collection locality in this study. Its biology is unknown. However, specimens were collected in a VSR trap baited with decomposing chicken in the present study.

**28. Peckia (Peckia) chrysostoma (Wiedemann, 1830)**

**Literature records.**Lopes (1941; Dodge (1965b); Lopes (1969); Pape (1996); Buenaventura and Pape (2013); Mello-Patiu (2016); Dufek (2019); Dufek et al. (2020); Toma et al. (2020).

**Neotropical distribution.** American Virgin Is, Argentina, Bahamas, Belize, Bolivia, Brazil, Chile, Colombia, Costa Rica, Dominica, Ecuador, French Guiana, Galápagos Is, Guatemala, French Guiana, Guyana, Jamaica, Mexico, Nicaragua, Panama, Peru, Surinam, Trinidad & Tobago, Venezuela.

**Newly collected material.** • Rio Bueno Property, St. Ann; 31 May 2018; 1 ♂; L. Foote and E. Buenaventura leg. (DLSUWI) • Belair, St. Ann; 31 May 2018; 2 ♂; L. Foote and E. Buenaventura leg. (DLSUWI) • Green Grotto, St. Ann; 31 May 2018; 3 ♂; L. Foote and E. Buenaventura leg. (DLSUWI) • Windsor, Trelawny; 01 Jun. 2018; 2 ♂; L. Foote and E. Buenaventura leg. (DLSUWI) • Bowden Pen, St. Thomas; 05 Jun. 2018; 5 ♂; L. Foote and E. Buenaventura leg. (DLSUWI) • Mona, St. Andrew; 17 Jun. 2018; 10 ♂; L. Foote leg. (DLSUWI) • Newport, Manchester; 18 Aug. 2023; 4 ♂; R. Daley leg. (DLSUWI) • Comfort Castle, Portland; 27 Mar. 2024; 2 ♂; L. Foote leg. (DLSUWI).

**Museum material.** • Copa Cabana, St. Thomas ; 24 Jan. 1989; 1 ♂; N. Knight leg. (DLSUWI) • Gordon Town, St. Andrew; 15 Jan. 2009; 1 ♂; J. Wynter leg. (DLSUWI) • May Pen, Clarendon; 21 Nov. 2010; 1 ♂; T. Gooden leg. (DLSUWI) • Guys Hill, St. Catherine; 23 Nov. 2011; 2 ♂; D. Allen leg. (DLSUWI) • Green Grotto, St. Ann; 13 Nov. 2010; 1 ♂; D. Herro leg. (DLSUWI) • Discovery Bay, St. Ann; 14 Sept. 2014; 1 ♂; J. Dixon leg. (DLSUWI) • Windsor, Trelawny; 04 Oct. 2014; 1 ♂; Hanchard leg. (DLSUWI) • Mona, St. Andrew; 23 Sept. 2014; 3 ♂; R. Daley leg. (DLSUWI) • Roaring River, St. Ann; 05 Nov. 2016; 1 ♂; S. McKenzie leg. (DLSUWI).

**Remarks.**Peckia (Peckia) chrysostoma is one of the most widely distributed species in the genus *Peckia* (Buenaventura and Pape 2013). It has been reported as a coloniser of human corpses in Brazil (Vasconcelos et al. 2014), and Dodge (1965b) mentions specimens from Jamaica that were “bred from crocodile”. Specimens have been recorded as collected from stinkhorn fungus (*Phallus* sp.; Phallales: Basidiomycota) and flowers of *Casearia* sp. (Salicaceae) (Camargo et al. 2018).

**29. Peckia (Sarcodexia) dominicana (Lopes, 1982)**

Fig. 5

**Figure 5.**
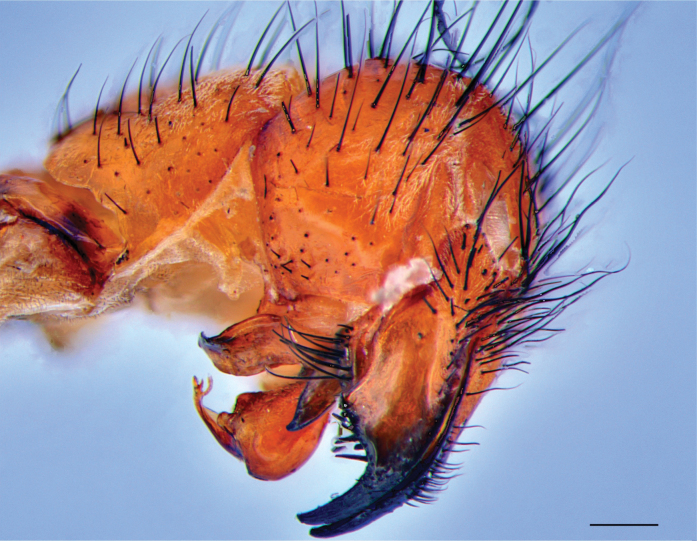
Peckia (Sarcodexia) dominicana. Male terminalia, postero-lateral view; Antillean species, new record from Jamaica. Scale bar: 1 mm.

**Neotropical distribution.** Dominican Republic, Jamaica (new record).

**Newly collected material.** • Windsor, Trelawny; 01 Jun. 2018; 1 ♂; L. Foote and E. Buenaventura leg. (DLSUWI) • Hardware Gap, Portland; 29 May 2018; 2 ♂; L. Foote and E. Buenaventura leg. (DLSUWI) • Red Light, St. Andrew; 20 Mar. 2024; 1 ♂; L. Foote leg. (DLSUWI).

**Remarks.** Previously known only from the Dominican Republic. This study reports Peckia (Sarcodexia) dominicana as a new record for Jamaica and adds to its distribution within the Caribbean. Little is known about the biology of *P.dominicana*. It was collected in a VSR trap baited with decomposing chicken in the present study.

**30. Peckia (Peckia) hillifera (Aldrich, 1916)**

**Literature records.**Buenaventura and Pape (2013); (Camargo et al. 2018).

**Neotropical distribution.** Bahamas, Brazil, Cuba, Jamaica, México, Panamá, Puerto Rico, Trinidad & Tobago, Venezuela.

**Newly collected material.** • Belair, St. Ann; 31 May 2018; 1 ♂; L. Foote and E. Buenaventura leg. (DLSUWI).

**Remarks.** Specimens have been reared from a dead crab [*Ucidescordata* (Linnaeus)] (Camargo et al. 2018).

**31. Peckia (Sarcodexia) lambens (Wiedemann, 1830)**

**Literature records.**Townsend (1892, 1993, both as *Sarcodexiasternodontis*); Johnson (1908, 1919, both as *Sarcophagasternodontis*); Lopes (1969, as *Sarcodexiasternodontes*); Pape (1996); Buenaventura and Pape (2013); Vairo et. al (2011); Vairo et al. (2014); Mello-Patiu (2016); Dufek (2019); Dufek et al. (2020); Ramírez-Mora et al. (2022).

**Neotropical distribution.** Argentina, Bahamas, Bolivia, Brazil, Cayman Is, Chile, Colombia, Costa Rica, Cuba, Ecuador, El Salvador, Galápagos Is, Guadeloupe, Guyana, Haití, Honduras, Jamaica, México, Panamá, Paraguay, Perú, Puerto Rico, St. Vincent and the Grenadines, Trinidad & Tobago, Venezuela.

**Newly collected material.** • Green Grotto, St. Ann; 31 May 2018; 3 ♂; L. Foote and E. Buenaventura leg. (DLSUWI) • Belair, St. Ann; 31 May 2018; 2 ♂; L. Foote and E. Buenaventura leg. (DLSUWI) • Red Light, St. Andrew; 26 Feb. 2024; 3 ♂; L. Foote leg. (DLSUWI) • Newport, Manchester; 18 Aug. 2023; 2 ♂; R. Daley leg. (DLSUWI) • Comfort Castle, Portland; 27 Mar. 2024; 2 ♂; L. Foote leg. (DLSUWI).

**Museum material.** • Cambridge District, St. Elizabeth; 23 Nov. 2013; 1 ♂; Bailey leg. (DLSUWI) • August Town, St. Andrew; 09 Nov. 2017; 1 ♂; Dacosta leg. (DLSUWI).

**Remarks.** Known as a saprophagous and necrophagous species in the Neotropics (Lopes de Carvalho and Linhares 2001; Vairo et al. 2015). It has been reported on human corpses and is considered one of the most important saprophagous species of forensic importance (Vairo et al. 2015). It has been collected from decomposing fish, bovine spleen and faeces (Barbosa 2019). Known parasitoid of the yellowtail moth (*Hylesiametabus*) and the fall armyworm (*Spodopterafrugiperda*) (Toma et al. 2018).

**32. Peckia (Euboettcheria) nicasia (Lopes, 1941)**

**Literature records.**Dodge (1965b); Lopes (1941, 1969); Pape (1996); Buenaventura and Pape (2013).

**Neotropical distribution.** Jamaica.

**Newly collected material.** • Windsor, Trelawny; 01 Jun. 2018; 3 ♂; L. Foote and E. Buenaventura leg. (DLSUWI) • Green Grotto, St. Ann; 31 May 2018; 1 ♂; L. Foote and E. Buenaventura leg. (DLSUWI) • Hardware Gap, Portland; 29 May 2018; 2 ♂; L. Foote and E. Buenaventura leg. (DLSUWI) • Bowden Pen, St. Thomas; 05 Jun. 2018; 1 ♂; L. Foote and E. Buenaventura leg. (DLSUWI) • Mona, St. Andrew; 12 Jun. 2018; 2 ♂; L. Foote leg. (DLSUWI) • Red Light, St. Andrew; 20 Mar. 2024; 5 ♂; L. Foote leg. (DLSUWI).

**Museum material.** • Cinchona Morce’s Gap, St. Andrew; 21 Aug. 1949; 1 ♀; R. B. Bengry & R. Hart leg. (NHMJ) • Hermitage Reservoir, St. Andrew; 30 May 1954; 1 ♂; T. H. Farr leg. (NHMJ) • Southwest of Ecclesdown, Portland; 24 Aug. 1954; 1 ♂; T. H. Farr leg. (NHMJ) • Fern Gully, St. Ann; 11 Jul. 1954; 1 ♂; T. H. Farr leg. (NHMJ) • Hermitage Dam, St. Andrew; 31 May 1954; 1 ♂; R. B. Bengry leg. (NHMJ) • Long Mountain, St. Andrew; 26 Jun. 1955; 1 ♂; T. H. Farr leg. (NHMJ) • Benson Avenue; 12 Sep. 2007; 1 ♂; A. Sherman leg. (DLSUWI) • Bowden Pen, St. Thomas; 04 Nov. 2011; 1 ♂; T. Stephenson; (DLSUWI) • Roaring River, St. Ann; 03 Oct. 2014; 2 ♂; Bennett leg. (DLSUWI) • Dolphin Head Mountain, Hanover; 01 Oct. 2014; 1 ♀; L. Wright leg. (NHMJ).

**Remarks.** The species was collected in a VSR trap baited with decomposing chicken during the present study. It has previously been collected from decomposing pig carrion (Foote 2014).

##### Genus *Ravinia* Robineau-Desvoidy, 1863

**33. *Raviniaeffrenata* (Walker, 1861)**

**Literature records.**Johnson (1919; as Sarcophaga (Ravinia) quadrisetosa, see Dodge 1965b); Hall (1928, as *Sarcophagaadamsii*); Lopes (1969, as *Chaetoraviniaadamsi*); Pape (1996); Mello-Patiu (2016); Ramírez-Mora et al. (2022).

**Neotropical distribution.** Bahamas, Brazil, Colombia, Costa Rica, Cuba, Dominica, Dominican Republic, El Salvador, Guatemala, Jamaica, México, Panamá, Perú, St. Vincent.

**Newly collected material.** • Rio Bueno Property, St. Ann; 31 May 2018; 6 ♂; L. Foote and E. Buenaventura leg. (DLSUWI) • Belair, St. Ann; 31 May 2018; 2 ♂; L. Foote and E. Buenaventura leg. (DLSUWI) • Newport, Manchester; 18 Aug. 2023; 2 ♂; R. Daley leg. (DLSUWI).

**Museum material.** • Amity Hall, St. Catherine; 23 Mar. 1947; 1 ♂; C. B. Thompson leg. (NHMJ) • West of Jacob’s Hut, Clarendon; 28 Sept. 1954; 1 ♂; T. H. Farr leg. (NHMJ).

**Remarks.** Species collected from decomposing fish (sardines), human faeces (Barbosa 2019) and fruit (Valverde-Castro et al. 2017).

##### Genus *Sarcodexiopsis* Townsend, 1917

**34. *Sarcodexiopsiswelchi* (Hall, 1930)**

Fig. 6

**Figure 6.**
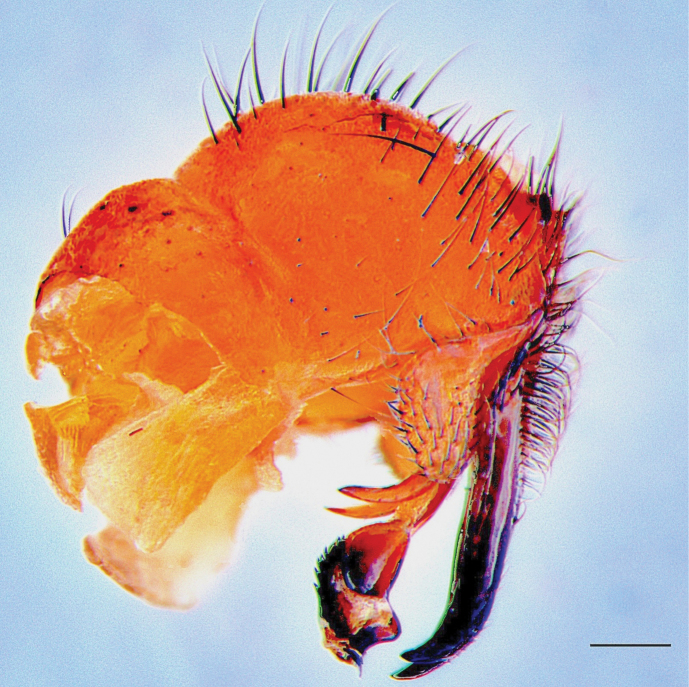
*Sarcodexiopsiswelchi*. Male terminalia, lateral view; Antillean species. Scale bar: 1 mm.

**Literature records.**Pape (1996).

**Neotropical distribution.** Bahamas, British Virgin Is, Cuba, Jamaica, Puerto Rico.

**Newly collected material.** • Belair, St. Ann; 31 May 2018; 1 ♂; L. Foote and E. Buenaventura leg. (DLSUWI).

##### Genus *Sarcofahrtiopsis* Hall, 1933

**35. *Sarcofahrtiopsisdiembroma* Dodge, 1965**

**Literature records.**Dodge (1965b); Lopes (1969); Pape (1996).

**Neotropical distribution.** Jamaica.

**Remarks.** This species is still known only from the original type series consisting of two females. The holotype from Second Breakfast Spring, St. Andrew (deposited in Washington State University), and a female paratype from Hermitage, St. Andrew, stated to be in the “Science Museum, Institute of Jamaica” (now Natural History Museum of Jamaica) but were not located.

**36. *Sarcofahrtiopsisfarri* Dodge, 1965**

**Literature records.**Dodge (1965b); Lopes (1969); Pape (1996); Pape and Méndez (2004).

**Neotropical distribution.** Costa Rica, Jamaica.

**Newly collected material.** • Green Grotto, St. Ann; 31 May 2018; 3 ♂; L. Foote and E. Buenaventura leg. (DLSUWI) • Belair, St. Ann; 31 May 2018; 3 ♂; L. Foote and E. Buenaventura leg. (DLSUWI) • Rio Bueno Property, St. Ann; 31 May 2018; 2 ♂; L. Foote and E. Buenaventura leg. (DLSUWI).

**Museum material.** • Ferry, St. Andrew; 03 Oct. 1954; 1 ♂; T. H. Farr leg. (NHMJ) • Rio Cobre, St. Catherine; 05 Jun. 1952; 1 ♂; R. P. Bengry leg. (NHMJ) • Colonel Ridge, Clarendon; 18 Nov. 1946; 1 ♂; G. B. Thompson leg. (NHMJ).

**37. *Sarcofahrtiopsisjamaicensis* Dodge, 1965**

**Literature records.**Dodge (1965b); Lopes (1969); Pape (1996).

**Neotropical distribution.** Jamaica.

**Museum material.** • Hermitage Dam, St. Andrew; 03 May 1954; 1 ♂; R. P. Bengry leg. (NHMJ).

**38. *Sarcofahrtiopsispaterna* Dodge, 1965**

**Literature records.**Dodge (1965b); Pape and Méndez (2004).

**Neotropical distribution.** Cuba, Jamaica, Puerto Rico.

**Remarks.** The presence of this species in Jamaica is based on one male paratype with no further data (Dodge 1965b).

##### Genus *Sarcophaga* Meigen, 1826

**39. Sarcophaga (Liopygia) ruficornis (Fabricius, 1794)**

**Literature records.**Pape (2024).

**Neotropical distribution.** Brazil, Colombia, Jamaica, Panamá, Venezuela.

**Newly collected material.** • Mona, St. Andrew; 07 Sep. 2018; 1 ♂; L. Foote leg. (DLSUWI).

**Remarks.** This is the first record from Jamaica documented with explicit reference to a collected specimen. Considered to be synanthropic and of forensic relevance (Barbosa 2019). Larvae were found to cause myiasis in toads (*Bufomelanostictus* Schneider) (Roy and Dasgupta 1977). It has been collected from decomposing bovine spleen and fish (Barbosa 2019) as well as from human cadavers (Kavitha et al. 2013). The optimum temperature for the development of *S.ruficornis* larvae is 20–35 °C (Nassu et al. 2014).

##### Genus *Tapacura* Tibana & Lopes, 1985

**40. *Tapacuramariarum* Tibana & Lopes, 1985**

Fig. 7

**Figure 7.**
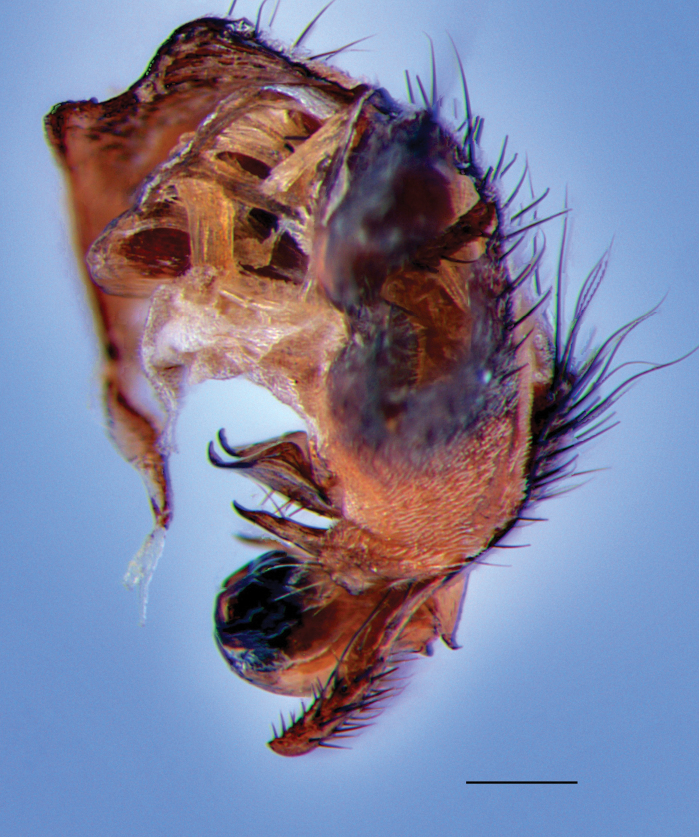
*Tapacuramariarum*. Male terminalia, lateral view; new record from Jamaica. Scale bar: 1 mm.

**Neotropical distribution.** Brazil, Jamaica (new record).

**Newly collected material.** Green Grotto, St. Ann; 31 May 2018; 4 ♂; L. Foote and E. Buenaventura leg. (DLSUWI).

**Remarks.** New record of this genus and species from Jamaica. The genus *Tapacura* presently contains two species, *Tapacuramariarum* recorded in the Neotropics (Brazil) and *Tapacuramexicana* Lopes, 1988 known only from the Nearctic (México) (Mello-Patiu and de Souza Neto 2007). There is no information on the biology of the species. It was collected from a VSR trap baited with decomposing chicken in the present study.

##### Genus *Titanogrypa* Townsend, 1860

**41. Titanogrypa (Airypel) cryptopyga Lopes, 1956**

**Literature records.**Dodge (1965b, as *Airypelmolluscoperda*); Lopes (1969); Pape (1996); Dufek (2019); Dufek et al. (2020).

**Neotropical distribution.** Argentina, Bolivia, Brazil, Cuba, Guyana, Jamaica, Trinidad & Tobago.

**Museum material.** • Ferry, St. Andrew; 03 Oct. 1954; 1 ♂; T. H. Farr leg. (NHMJ).

**Remarks.** Probably a scavenger. Dodge (1965b) gave label data from the holotype and a paratype: “Emerged Nov. 8, 1902, in Pittsburgh, Pa. Pupae received with shells received from near Mandeville, Jamaica”.

**42. Titanogrypa (Sarconeiva) fimbriata (Aldrich, 1916)**

**Literature records.**Johnson (1919); Dodge (1965b); Lopes (1969); Pape (1996); Vairo et. al (2011); Dufek (2019); Dufek et al. (2020).

**Neotropical distribution.** Argentina, Brazil, Dominica, Dominican Republic, Jamaica, México, Panamá, Perú, Venezuela.

**Newly collected material.** • Bowden Pen, St. Thomas; 05 Jun. 2018; 4 ♂; L. Foote and E. Buenaventura leg. (DLSUWI).

**Museum material.** • Mona, St. Andrew; 07 May 1989; 1 ♂; J. Lawrence leg. (DLSUWI) • Mona, St. Andrew; 17 Nov. 2009; 1 ♂; T. Henry leg. (DLSUWI).

**Remarks.** Considered to be of forensic relevance (Barbosa 2019). It has been recorded from decomposition studies in Brazil (Mello-Patiu et al. 2014), on gastropods/molluscs (Barker 2004), and decomposing sardines (Barbosa 2019).

##### Genus *Tricharaea* (Sarcophagula) Wulp, 1887

**43. *Tricharaeacanuta* (Wulp, 1896)**

**Literature records.**Dodge (1965b); Lopes (1969); Pape (1996); Mello-Patiu (2016); Ramírez-Mora et al. (2022).

**Neotropical distribution.** Brazil, Colombia, Costa Rica, Cuba, Dominica, Ecuador, El Salvador, Galápagos Is, Guatemala, Honduras, Jamaica, México, Paraguay, Perú.

**Newly collected material.** • Mona, St. Andrew; 07 Sep. 2018; 1 ♂; L. Foote leg. (DLSUWI).

**Remarks.** Synanthropic species of forensic relevance (Barbosa 2019). It has been collected from human faeces and decomposing bovine spleen (Barbosa 2019).

**44. Tricharaea (Sarothromyia) femoralis (Schiner, 1868)**

**Literature records.**Johnson (1908, 1919).

**Neotropical distribution.** Bahamas, Brazil, Costa Rica, Cuba, Dominica, French Guiana, Honduras, Panama, Puerto Rico, Surinam, Trinidad & Tobago, Turks & Caicos Is., Venezuela.

**Museum material.** • Holland Bay, St. Thomas; 16 Mar. 1989; 8 ♀; T. H. Farr leg. (NHMJ) • Holland Bay, St. Thomas; 16 Nov. 1988; 5 ♀; R. A. Boothe leg. (NHMJ).

**45. Tricharaea (Sarcophagula) occidua (Fabricius, 1794)**

**Literature records.**Johnson (1908, as *Sarcophagulaimbecilla*; 1919); Dodge (1965b); Dufek et al. (2020).

**Neotropical distribution.** American Virgin Is., Argentina, Bolivia, Brazil, Chile, Colombia, Cuba, Dominica, Ecuador, El Salvador, Galápagos Is, Guatemala, Guyana, Haiti, Honduras, Jamaica, Mexico, Panama, Paraguay, Peru, Puerto Rico, St. Vincent Is., Venezuela.

**Museum material.** • Swamp, St. Thomas; 03 Nov. 1955; 1 ♀; T. H. Farr leg. (NHMJ) • Half Way Tree, St. Andrew; 06 Aug. 1950; 2 ♀; R. B. Bengry leg. (NHMJ) • Windsor Hotel, St. Ann; 20 Sep. 1959; 1 ♀; T. H. Farr leg. (NHMJ) • Ferry, St. Andrew; 03 Oct. 1954; 1 ♀; T. H. Farr leg. (NHMJ).

**Remarks.** Only females were studied in the present study, and their separation from *T.canuta* (Wulp, 1896) remains tentative.

## ﻿Discussion

The updated checklist for Jamaica includes 45 species, four of which are new records. The number of genera in Jamaica has increased to 21 with the addition of the genera *Bahamiola* and *Tapacura*.

With the addition of Peckia (Sarcodexia) dominicana to the checklist, *Peckia* becomes the most speciose flesh fly genus in Jamaica with a total of six species: *P.buethni*, *P.chrysostoma*, *P.dominicana*, *P.hillifera*, *P.lambens*, and *P.nicasia*. Some species of *Peckia* were quite rare. Only one individual of *P.buethni* was collected in this study. Previous record of *P.buethni* was one male in Papine, St. Andrew (southern Jamaica), collected by W. Büthn (BMNH). Similarly, only one individual of *P.hillifera* was collected in this study. Previous record of *P.hillifera* was one male in Milk River bath, St. Thomas (southern Jamaica), collected by Wirth and Farr (ZMUC). This pattern suggests that *P.buethni* and *P.hillifera* are rare in Jamaica, despite their relatively wide distribution.

LepidodexiasubgenusHarpagopyga Aldrich contains 15 nominal species, 14 of which occur in the Neotropical region (Pape 1996). Dodge (1965b) documented five species of *Lepidodexia* from Jamaica, all of which are endemic to the island. An additional species, *L.diversipes*, is here added to the records of *Lepidodexia* from Jamaica, increasing the total species number to six. Of note, no specimens collected during the present study, suggesting low abundance, a very narrow distribution, or sparse collecting for flesh flies in Jamaica.

*Oxysarcodexia* consists of 91 described species worldwide and is considered one of the most species-rich genera in the Neotropics (Souza et al. 2020). Jamaica has five species of *Oxysarcodexia*, making it one of the most speciose genera on the island after *Peckia* and *Lepidodexia*. There are two endemic species of *Oxysarcodexia* recorded for Jamaica: *O.corolla* and *O.dorisae*. Only the female of *O.dorisae* is known, while both the male and the female of *O.corolla* are known. *Oxysarcodexiacorolla* was found in a wet limestone forest, wet forest, and a rural area in St. Andrew, which might indicate a preference for environments with low anthropogenic impact. All other known species of *Oxysarcodexia* in Jamaica are widely distributed.

*Bahamiolaorbitalis* was previously known only from the Bahamas (Grand Bahama Is.; Dodge 1965a). With 94 individuals across five locations, the species is common and widely distributed (Table 4).

*Tapacuramariarum* was previously known only from Brazil (Tibana and Lopes 1985), and the present record represents a significant range extension. Four individuals were collected at the Green Grotto, St. Ann. This species is likely to have a restricted geographical range in Jamaica, and its occurrence at a single locality may suggest a limited distribution in the island.

**Figure 8.**
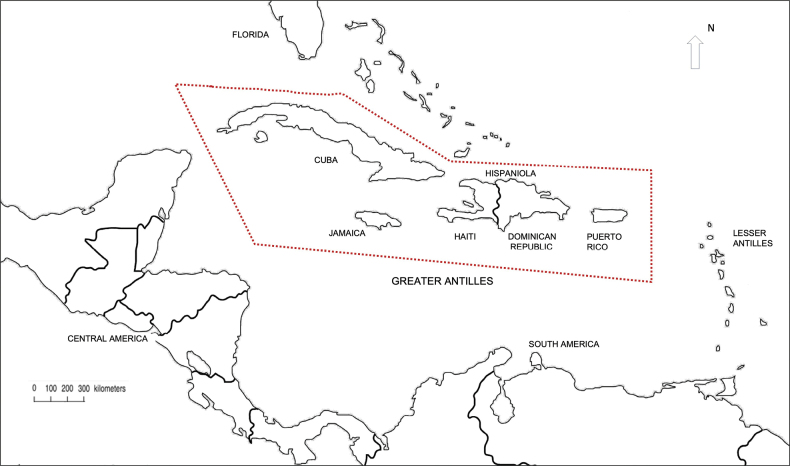
Map of the Caribbean region, highlighting the Greater Antilles.

MacArthur and Wilson (1967) demonstrated that the number of species on an island is correlated with its size and proximity to the mainland. The Caribbean islands share several species due to their proximity and shared geological histories. According to Crews and Esposito (2020) islands are sources of diversity with dispersal from a large island source to smaller islands. Notably, Cuba, the largest island of the Greater Antilles (Fig. 8, Table 4), has the largest number of known species of Sarcophagidae. There are 15 species shared between Jamaica and Cuba, which may be attributed mainly to their close proximity, as Jamaica is approximately 145 km from the southeastern coast of Cuba. Winds may further facilitate species dispersal between these islands (Kirk-Spriggs and Muller 2017).

Hispaniola is situated 190 km east of Jamaica. A total of 19 species of Sarcophagidae have been identified on the island, and of these, eight species are shared with Jamaica. It is noteworthy that Hispaniola is approximately seven times larger than Jamaica (Table 4), suggesting that Hispaniola may be under-sampled or inadequately studied. Another factor suggesting low sampling efforts on the island of Hispaniola is the low number of species shared between the two countries of the island. Eight species are recorded from the Haitian part and 13 from the Dominican Republic. Only two species are found in both countries, indicating inadequate sampling.

Puerto Rico, the smallest island in the Greater Antilles (Table 4), is the furthest from Jamaica, located at a distance of 923 km. Despite this distance, Puerto Rico and Jamaica share 12 species, which may reflect extensive sampling efforts in Puerto Rico.

Several species previously thought to be endemic to other islands have been found in Jamaica. It is unclear whether these species were recently introduced to Jamaica or if their endemism to other islands was mistakenly identified. A genetic analysis of these populations is needed to resolve these uncertainties.

Compared to other islands in the Greater Antilles, Jamaica is notable for its high endemism of Sarcophagidae. With an area of 10,992 km^2^, Jamaica is the third largest island in the Greater Antilles (Fig. 8). The island’s diverse geography, which includes complex topography such as extensive karst limestone regions, mountains and plains, along with a range of biomes from xerophytic conditions receiving less than 60 cm of annual precipitation to wet forests receiving more than 700 cm, has fostered numerous centres of speciation (Aitken-Soux et al. 1981), contributing to its high endemism. Specific regions, such as the Cockpit Country, are known to be local centres of endemism due to their distinctive geomorphology, characterized by isolated conical hills and depressions (Sweeting 1958), which limit species dispersal and create distinct microhabitats.

**Table 4.** Total number of endemics and percentage endemism of Sarcophagidae known from islands of the Greater Antilles.

Island	Number of endemics	Percentage of endemics (%)	Number of species	Size of island (km^2^)
Jamaica	15	33	45	10,992
Cuba	14	25	55	109,884
Hispaniola	3	14	19	76,192
Puerto Rico	4	13	30	8,870

## ﻿Additional information

### Author ORCIDs

Latoya Foote-Gordon https://orcid.org/0009-0001-6713-0550

Eric Garraway https://orcid.org/0009-0004-5586-1649

Thomas Pape https://orcid.org/0000-0001-6609-0609

Eliana Buenaventura https://orcid.org/0000-0002-5265-815X

### Data availability

All of the data that support the findings of this study are available in the main text.

## References

[B1] Aitken-Soux P Wahab AH Johnson IE (1981) Country-level action plan- Jamaica. IICA Biblioteca, Venezuela, 18 pp.

[B2] Bänziger H Pape T (2004) Flowers, faeces and cadavers: natural feeding and laying habits of flesh flies in Thailand (Diptera: Sarcophagidae, *Sarcophaga* spp.). Journal of Natural History 38(13): 1677–1694. 10.1080/0022293031000156303

[B3] Barbosa TM (2019) Sarcophagidae (Diptera) no bioma caatinga: revisão taxonômica do subgênero Titanogrypa (Cucullomyia) e avaliação do potencial de espécies como bioindicadoras de conservação ambiental. PhD Thesis, Universidade Federal de Pernambuco, Recife, Brazil. https://repositorio.ufpe.br/handle/123456789/34067

[B4] Barker GM (2004) Natural enemies of terrestrial molluscs. CABI Publishing, Wallingford, Oxfordshire, 644 pp. 10.1079/9780851993195.0085

[B5] Barros RM Mello-Patiu CA Pujol-Luz JR (2008) Sarcophagidae (Insecta, Diptera) associated to the decay process of *Susscrofa* Linnaeus (Suidae) carcasses in a cerrado area of distrito federal, Brazil. Revista Brasileira de Entomologia 52(4): 606–609. 10.1590/S0085-56262008000400011

[B6] Beltran Y Pinilla T Segura NA Bello FJ (2012) Synanthropy of Calliphoridae and Sarcophagidae (Diptera) in Bogotá, Colombia. Neotropical Entomology 41(3): 237–242. 10.1007/s13744-012-0036-x 23950049

[B7] Buenaventura E (2021) Museomics and phylogenomics with protein-encoding ultraconserved elements illuminate the evolution of life history and phallic morphology of flesh flies (Diptera: Sarcophagidae). BMC Ecology and Evolution 21(70): 1–18. 10.1186/s12862-021-01797-7 33910519 PMC8082969

[B8] Buenaventura E Pape T (2013) Revision of the new world genus *Peckia* Robineau-Desvoidy (Diptera: Sarcophagidae). Zootaxa 3622(1): 1–87. 10.11646/zootaxa.3622.1.1 25320760

[B9] Buenaventura E Valverde-Castro C Wolff M (2021a) New carrion-visiting flesh flies (Diptera: Sarcophagidae) from tropical dry forests of Colombia and their phylogenetic affinities. Acta Tropica 213: 105720. 10.1016/j.actatropica.2020.105720 33007304

[B10] Buenaventura E Lloyd MW Perilla-López JM González VL Thomas-Cabianca A Dikow T (2021b) Protein-encoding ultraconserved elements provide a new phylogenomic perspective of Oestroidea flies (Diptera: Calyptratae). Systematic Entomology 46: 5–27. 10.1111/syen.12443

[B11] Camargo SLX Carvalho-Filho FS Esposito CM (2018) The genus *Peckia* Robineau-Desvoidy (Diptera: Sarcophagidae) in the Brazilian Amazon: a new species, new records, descriptions of female terminalia and key to species. Zootaxa 4483(1): 1–35. 10.11646/zootaxa.4483.1.1 30313797

[B12] Carvalho LML Linhares AX (2001) Seasonality of insect succession and pig carcass decomposition in a natural forest area in south-eastern Brazil. Journal of Forensic Science 46(3): 604–608. 10.1520/JFS15011J 11372997

[B13] Carvalho-Filho FS Esposito CM (2012) Revision of *Argoravinia* Townsend (Diptera: Sarcophagidae) of Brazil with the description of two new species. Zootaxa 3256: 1–26. 10.11646/zootaxa.3256.1.1

[B14] Cherix D Wyss C Pape T (2012) Occurrences of flesh flies (Diptera: Sarcophagidae) on human cadavers in Switzerland, and their importance as forensic indicators. Forensic Science International 220(1–3): 158–163. 10.1016/j.forsciint.2012.02.016 22465019

[B15] Crews SC Esposito LA (2020) Towards a synthesis of the Caribbean biogeography of terrestrial arthropods. BMC Ecology Biology 20: 12. 10.1186/s12862-019-1576-z PMC6979080 31980017

[B16] Curran CH (1928) Insects of Porto Rico and the Virgin Islands. Diptera or two-winged flies. Scientific Survey of Porto Rico and the Virgin Islands 11: 1–118.

[B17] Dahlem G Downes Jr W (1996) Revision of the genus *Boettcheria* in America North of Mexico (Diptera: Sarcophagidae). Insecta Mundi: A Journal of World Insect Systematics 10(1–4): 77–103. https://digitalcommons.unl.edu/cgi/viewcontent.cgi?article=1361&context=insectamundi

[B18] Dodge RH (1965a) The Sarcophagidae (Diptera) of the West Indies. I. The Bahamas Islands. Annals of the Entomological Society of America 58(4): 474–497. 10.1093/aesa/58.4.474

[B19] Dodge RH (1965b) The Sarcophagidae (Diptera) of the West Indies II. Jamaica. Annals of the Entomological Society of America 58(4): 497–517. 10.1093/aesa/58.4.497

[B20] Dufek MI (2019) Comunidades de Calliphoridae y Sarcophagidae (Diptera: Calyptrate) en areas naturales y disturbados del Chaco Oriental. PhD Thesis, Universidad Nacional del Nordeste, Argentina, 179 pp.

[B21] Dufek MI Osherov EB Mulieri PR (2015) Preliminary survey and inventory of Calliphoridae and Sarcophagidae (Diptera) in the Province of Corrientes, Argentina, with new records of species with forensic importance. Revista de La Sociedad Entomológica Argentina 74(1–2): 37–46. https://ri.conicet.gov.ar/handle/11336/208116

[B22] Dufek MI Mello-Patiu CA Mulieri PR (2020) Inventory of Sarcophaginae (Diptera: Sarcophagidae) for the Humid Chaco, a poorly surveyed ecoregion of South America. Journal of Natural History 54(5–6): 367–403. 10.1080/00222933.2020.1764646

[B23] Early M Goff ML (1986) Arthropod succession patterns in exposed carrion on the island of O’ahu, Hawaiian islands, USA. Journal of Medical Entomology 23(5): 520–531. 10.1093/jmedent/23.5.520 3772956

[B24] Ferrar P (1987) A guide to the breeding habits and immature stages of DipteraCyclorrhapha. Entomonograph 8(1–2): 1–907. 10.1163/9789004533936

[B25] Flores VI Dale WE (1995) Un estudio sobre la ecología de las moscas Sarcophagidae en la costa central Peruana. Revista Peruana de Entomología 38: 13–17.

[B26] Foote L (2014) An introduction to the study of insects of forensic importance in Jamaica. Master’s Thesis, University of the West Indies, Mona, Jamaica.

[B27] Foote-Gordon L Garraway E (2023a) Ultrastructure Morphology of the Antennae of *Bahamiolaorbitalis* (Diptera: Sarcophagidae). Caribbean Journal of Science 53(1): 51–58. 10.18475/cjos.v53i1.a5

[B28] Foote-Gordon L Garraway E (2023b) Ultrastructure morphology of the antennae of three *Peckia* species; *Peckiadominicana*, *P.nicasia*, *P.chrysostoma* (Diptera: Sarcophagidae). Caribbean Journal of Science 53(2): 198–209. 10.18475/cjos.v53i2.a4

[B29] Foote-Gordon L Garraway E (2023c) Ultrastructure Morphology of the Antennae of *Oxysarcodexiacorolla* and *Oxysarcodexiapeltata* (Diptera: Sarcophagidae). Caribbean Journal of Science 53(2): 384–390. 10.18475/cjos.v53i2.a19

[B30] Freeman BE Jayasingh DB (1975) Population dynamics of *Pachodynerusnasidens* (Hymenoptera) in Jamaica. Oikos 26(1): 86–91. 10.2307/3543282

[B31] Freeman BE Taffe CA (1974) Population dynamics and nesting behaviour of *Eumenescolona* (Hymenoptera) in Jamaica. Oikos 25: 388–394. 10.2307/3543961

[B32] Giroux M Wheeler T (2009) Systematics and phylogeny of the subgenus Sarcophaga (Neobellieria) (Diptera: Sarcophagidae). Annals of the Entomological Society of America 102(4): 567–587. 10.1603/008.102.0401

[B33] Gowdey CC (1926) Catalogus Insectorum Jamaicensis. Entomological Bulletin 4: 1–114.

[B34] Grindley-Watson EJ (2004) Faunal succession of necrophilous insects on wildlife carcasses in Louisiana. Journal of Medical Entomology 40(3): 338–347. 10.1603/0022-2585-40.3.338 12943113

[B35] Hall DG (1928) *Sarcophagapallinervis* and related species in the Americas. Annals of the Entomological Society of America 21: 331–352[, 4 pls]. 10.1093/aesa/21.2.331

[B36] Howlett BG Davidson MM Pattemore DE Walker MK Nelson WR (2016) Seasonality of calliphorid and sarcophagid flies across Canterbury arable farms requiring pollinators. New Zealand Plant Protection 69: 290–295. 10.30843/nzpp.2016.69.5899

[B37] Hwang C Turner BD (2005) Spatial and temporal variability of necrophagous Diptera from urban to rural areas. Medical and Veterinary Entomology 19(4): 379–391. 10.1111/j.1365-2915.2005.00583.x 16336303

[B38] Johnson CW (1908) The Diptera of the Bahamas, with notes and description of one new species. Psyche 15: 69–80. 10.1155/1908/81853

[B39] Johnson CW (1919) A revised list of Diptera of Jamaica. Bulletin Museum of Natural History 41: 441–449. http://hdl.handle.net/2246/1354

[B40] Kavitha R Nazni WA Tan TC Lee HL Azirun MS (2013) Review of forensically important entomological specimens collected from human cadavers in Malaysia (2005–2010). Journal of Forensic and Legal Medicine 20(5): 480–482. 10.1016/j.jflm.2013.03.007 23756518

[B41] Kirk-Spriggs AH Muller BS (2017) Biogeography of Diptera. Manual of Afrotropical Diptera 1: 203–238.

[B42] Livingstone SR (2006) Sea turtle ecology and conservation on the north coast of Trinidad, West Indies. PhD Thesis, University of Glasgow, Scotland, 263 pp. https://theses.gla.ac.uk/4323/

[B43] Lopes HS (1941) Sôbre alguns sarcofagídeos neotrópicos da coleção do Museu Britânico (Diptera). Arquivos de Zoologia, São Paulo 2(16): 357–388. 10.11606/issn.2176-7793.19412357-388

[B44] Lopes HS (1946) Contribuição ao conhecimento das espécies do gênero *Oxysarcodexia* Townsend, 1917 (DipteraSarcophagidae). Boletin de la Escuela Nacional de Veterinaria (Rio de Janeiro) 1: 62–134.

[B45] Lopes HS (1969) Family Sarcophagidae. In: Papavero N (Ed.) A catalog of the Diptera of the Americas south of the United States 103: 1–88. Departamento de Zoologia, Secretaria da Agricultura, São Paulo.

[B46] Lopes HS (1982) On *Eumacronychiasternalis* Allen (Diptera, Sarcophagidae), with larvae living on eggs and hatchilings [sic] of the East Pacific Green Turtle. Revista Brasileira de Biologia 42: 425–429.

[B47] MacArthur RH Wilson EO (1967) The theory of island biogeography. Princeton University Press, 224 pp.

[B48] Mello CA (1996) Revision of the genus *Farrimyia* Dodge, 1965 (Diptera, Sarcophagidae) – Parte I. Revista brasileira de Biologia 56: 459–471.

[B49] Mello-Patiu CA (2016) Family Sarcophagidae. Zootaxa 4122(1): 884–903. 10.11646/zootaxa.4122.1.75 27395323

[B50] Mello-Patiu CA de Souza Neto SP (2007) Revisão das duas espécies de *Tapacura* Tibana & Lopes, 1985 (Diptera: Sarcophagidae: Sarcophaginae). Biota Neotropica 7(1): 1–4. 10.1590/S1676-06032007000100021

[B51] Mello-Patiu CA Paseto ML Faria LS Mendes J Linhares AX (2014) Sarcophagid flies (Insecta, Diptera) from pig carcasses in Minas Gerais, Brazil, with nine new records from the Cerrado, a threatened neotropical biome. Revista Brasileira de Entomologia 58(2): 142–146. 10.1590/S0085-56262014000200005

[B52] Mullen GR Trauth SE Sellers JC (1984) Association of a miltogrammine fly, *Eumacronychianigricornis* Allen (Diptera: Sarcophagidae), with the brood burrows of *Sceloporusundulatus* (Latrielle) [sic] (Reptilia: Lacertillia [sic]). Journal of the Georgia Entomological Society 19: 1–6.

[B53] Nassu MP Thyssen PJ Linhares AX (2014) Developmental rate of immatures of two fly species of forensic importance: Sarcophaga (Liopygia) ruficornis and *Microcerellahalli* (Diptera: Sarcophagidae). Parasitology Research 113(1): 217–222. 10.1007/s00436-013-3646-2 24189973

[B54] Oliveira TC Vasconcelos SD (2010) Insects (Diptera) associated with cadavers at the Institute of Legal Medicine in Pernambuco, Brazil: Implications for forensic entomology. Forensic Science International 198(1–3): 97–102. 10.1016/j.forsciint.2010.01.011 20181449

[B55] Pape T (1989) Revision of *Opsidia* Coquillett (Diptera: Sarcophagidae). Entomologica Scandinavica 20(2): 229–241. 10.1163/187631289X00302

[B56] Pape T (1996) Catalogue of Sarcophagidae of the World (Insecta: Diptera). Memoirs of Entomology International 8: 1–558.

[B57] Pape T (2024) Family: Sarcophagidae a taxonomic database to all flesh flies. https://diptera.dk/sarco/index.php [Accessed on 06 May 2024]

[B58] Pape T Méndez J (2004) Two new species of *Sarcofahrtiopsis* (Diptera: Sarcophagidae). Zootaxa 485: 1–7. 10.11646/zootaxa.485.1.1

[B59] Pape T Blagoderov V Mostovski MB (2011) Order Diptera Linnaeus, 1758. Zootaxa 3148: 222–229. 10.11646/zootaxa.3148.1.42

[B60] Ramírez-Mora MA Buenaventura E Gómez-P LM Amat E (2012) Updated checklist and new records of calyptratae carrion flies (Diptera, Schizophora) from Valle de Aburrá and other localities in Colombia. Entomotropica 27(1): 27–35. 10.22201/ib.20078706e.2012.2.983

[B61] Ramírez-Mora MA Durango-Manrique Y Gomez GF (2022) New records and distributional data of (Diptera: Sarcophaginae) from Colombia. Revista de la Sociedad Entomológica Argentina 81(2): 49–56. 10.25085/rsea.80205

[B62] Roy P Dasgupta B (1977) *Sarcophagaruficornis* Fabr. (Sarcophagidae: Diptera) as a parasite of the common indian toad. Proceedings of the Indian Academy of Sciences 86(3): 207–209. 10.1007/BF03050949

[B63] Sánchez-Núñez DA Mancera-Pineda JE (2012) Pollination and fruit set in the main neotropical mangrove species from the southwestern Caribbean. Aquatic Botany 103: 60–65. 10.1016/j.aquabot.2012.06.004

[B64] Segura NA Bonilla MA Usaquén W Bello García FJ (2011) Entomofauna resource distribution associated with pig cadavers in Bogotá DC. Medical and Veterinary Entomology 25(1): 46–52. 10.1111/j.1365-2915.2010.00933.x 21143612

[B65] Smith JM (2001) Glasgow University Exploration Society Trinidad Expedition 2001. http://www.glasgowexsoc.org.uk/reports/trinidad2001.pdf [Accessed 15 January 2024]

[B66] Sousa JRP Carvalho-Filho FS Esposito MC Meyer M (2015) Distribution and abundance of necrophagous flies (Diptera: Calliphoridae and Sarcophagidae) in Maranhão, Northeastern Brazil. Journal of Insect Science 15(1): 70. 10.1093/jisesa/iev054 26078304 PMC4648181

[B67] Souza CM Pape T Thyssen PJ (2020) *Oxysarcodexia* Townsend, 1917 (Diptera: Sarcophagidae)-a centennial conspectus. Zootaxa 4841(1): 1–126. 10.11646/zootaxa.4841.1.1 33056796

[B68] Sukontason KL Piangjai S Bunchu N Chaiwong T Sripakdee D Boonsriwong W Vogtsberger RC Sukontason K (2006) Surface ultrastructure of the puparia of the blow fly, *Luciliacuprina* (Diptera: Calliphoridae), and flesh fly, *Liosarcophagadux* (Diptera: Sarcophagidae). Parasitology Research 98(5): 482–487. 10.1007/s00436-005-0102-y 16416125

[B69] Sweeting MM (1958) The karstlands of Jamaica. The Geographical Journal 124(2): 184–199. 10.2307/1790245

[B70] Szpila K Mądra A Jarmusz M Matuszewski S (2015) Flesh flies (Diptera: Sarcophagidae) colonising large carcasses in Central Europe. Parasitology Research 114(6): 2341–2348. 10.1007/s00436-015-4431-1 25876045 PMC4430587

[B71] Tibana R Lopes HS (1985) On Brazilian Sarcophagidae (Diptera) with description of two new genera and four new species. Revista Brasileira de Entomologia 29(2): 189–198.

[B72] Toma R Roel A Miranda R (2018) First record of Peckia (Sarcodexia) lambens (Wiedemann, 1830) (Diptera: Sarcophagidae) parasitizing *Spodopterafrugiperda* (Smith, 1797) (Lepidoptera: Noctuidae) in Brazil. Arquivos do Instituto Biológico 84: e0302016. 10.1590/1808-1657000302016

[B73] Toma R Koller WW Mello-Patiu CA Mello RL (2020) New records of Sarcophagidae (Insecta: Diptera) collected in Cerrado fragments in the municipality of Campo Grande, Mato Grosso do Sul state, Brazil. EntomoBrasilis 13: e0873. 10.12741/ebrasilis.v13.e0873

[B74] Townsend CHT (1892) A dexiid parasite of a longicorn beetle. Journal of the Institute of Jamaica 1: 105–106.

[B75] Vairo KP Mello-Patiu CA Carvalho CJB (2011) Pictorial identification key for species of Sarcophagidae (Diptera) of potential forensic importance in southern Brazil. Revista Brasileira de Entomologia 55(3): 333–347. 10.1590/S0085-56262011005000033

[B76] Vairo KP Ururahy-Rodrigues A Moura MO Mello-Patiu CA (2014) Sarcophagidae (Diptera) with forensic potential in Amazonas: a pictorial key. Tropical Zoology 27(4): 140–152. 10.1080/03946975.2014.981482

[B77] Vairo KP Queiroz MMC Mendonca PM Barbosa RR Carvalho CJB (2015) Description of immature stages of the flesh fly Peckia (Sarcodexia) lambens (Wiedemann) (Diptera: Sarcophagidae) provides better resolution for taxonomy and forensics. Tropical Zoology 28(3): 114–125. 10.1080/03946975.2015.1057435

[B78] Valverde-Castro C Buenaventura E Sánchez-Rodríguez JD Wolff M (2017) Flesh flies (Diptera: Sarcophagidae: Sarcophaginae) from the Colombian Guajira biogeographic Province, an approach to their ecology and distribution. Zoologia 34: 1–11. 10.3897/zoologia.34.e12277

[B79] Vasconcelos SD Soares TF Costa DL (2014) Multiple colonization of a cadaver by insects in an indoor environment: first record of *Fanniatrimaculata* (Diptera: Fanniidae) and Peckia (Peckia) chrysostoma (Sarcophagidae) as colonizers of a human corpse. International Journal of Legal Medicine 128(1): 229–233. 10.1007/s00414-013-0936-2 24218014

[B80] Villet MH Clitheroe C Williams KA (2017) The temporal occurrence of flesh flies (Diptera, Sarcophagidae) at carrion-baited traps in Grahamstown, South Africa. African Invertebrates 58(1): 1–8. 10.3897/AfrInvertebr.58.9537

[B81] Wells JD Smith JL (2013) First report of *Blaesoxiphaplinthopyga* (Diptera: Sarcophagidae) from a human corpse in the U.S.A. and a new state geographic record based on specimen genotype. Journal of Forensic Sciences 58(5): 1378–1380. 10.1111/1556-4029.12246 23899435

[B82] Wilson A (2004) Jamaica the Land. Crabtree Publishing Company, New York, 32 pp.

[B83] Wisniewska N Lipinska MM Golebiowski M Kowalkowska AK (2019) Labellum structure of *Bulbophyllumechinolabium* JJ Sm. (section Lepidorhiza Schltr., Bulbophyllinae Schltr., Orchidaceae Juss.). Protoplasma 256: 1185–1203. 10.1007/s00709-019-01372-4 30993470 PMC6713679

[B84] Yan L Buenaventura E Pape T Kutty SN Bayless KM Zhang D (2021) A phylotranscriptomic framework for flesh fly evolution (Diptera, Calyptratae, Sarcophagidae). Cladistics 37: 540–558. 10.1111/cla.12449 34570937

[B85] Yepes-Gaurisas D Sánchez-Rodríguez JD Mello-Patiu CA Wolff ME (2013) Synanthropy of Sarcophagidae (Diptera) in La Pintada, Antioquia-Colombia. Revista de Biologia Tropical 61(3): 1275–1287. https://www.scielo.sa.cr/pdf/rbt/v61n3/a22v61n3.pdf 24027923

